# Changes in institution for mental diseases (IMD) ownership status and insurance acceptance over time

**DOI:** 10.1093/haschl/qxad089

**Published:** 2024-01-16

**Authors:** Mara A G Hollander, Alexandra Patton, Morgan C Shields

**Affiliations:** Department of Public Health Sciences, University of North Carolina at Charlotte,Charlotte, NC 28223, United States; Department of Public Health Sciences, University of North Carolina at Charlotte,Charlotte, NC 28223, United States; Brown School of Social Work, Washington University in St. Louis, St. Louis, MO 63130, United States

**Keywords:** institutions for mental disease, psychiatric hospital, hospital payment, Medicaid

## Abstract

State Medicaid programs are prohibited from using federal dollars to pay institutions for mental diseases (IMDs)—freestanding psychiatric facilities with more than 16 beds. Increasingly, regulatory mechanisms have made payment of treatment in these settings substantially more feasible. This study evaluates if changing financial incentives are associated with increases in for-profit ownership among IMD facilities relative to non-IMD facilities, as well as greater increases in Medicaid acceptance among for-profit IMD facilities relative to for-profit non-IMD facilities. We used data from the 2014–2020 National Mental Health Services Surveys and examined 11 945 facility-years. Relative to non-IMDs, the increase in for-profit ownership among IMDs was 6.6 percentage points greater. The largest proportional change in Medicaid acceptance occurred among for-profit IMD facilities relative to for-profit non-IMDs (18.5 percentage points). Existing research is mixed on the quality of inpatient and residential psychiatric care provided in for-profit vs nonprofit and public facilities, as well as in IMD relative to non-IMD facilities. As payment policy increasingly incentivizes for-profit facilities to enter the psychiatric care space, we should be mindful of the impact of these decisions on patient safety.

## Introduction

Since Medicaid's inception, state Medicaid programs have been prohibited from using matching federal dollars to pay for the treatment of beneficiaries ages 21–64 years in institutions for mental diseases (IMDs)—freestanding facilities with more than 16 beds providing treatment for mental illness or substance use disorders (SUDs).^1^

In inpatient and residential treatment settings, patients with acute concerns can be monitored 24/7, which may not be possible in community-based treatment settings like community mental health centers.^2^ Patients who exhibit self-harming or suicidal behaviors, or who present a danger to other people, are those most likely to be admitted.

Increasingly, states are using a variety of regulatory mechanisms to increase access to inpatient and residential beds, including, with federal encouragement, the submission of Section 1115 waivers: waivers to pay for mental health treatment in IMDs are approved in 11 states and pending in 6 others, while waivers to pay for SUD treatment have been accepted in 35 states.^3^ States may also allow Medicaid managed-care programs to offer treatment in IMD settings “in lieu of” treatment in other settings for up to 15 days per month, or use Disproportionate Share Hospital payments to pay for care, particularly if they treat forensic patients.^4-6^

The changing policy landscape for the payment of inpatient and residential mental health treatment is likely impacting the kinds of facilities in which patients receive care. Between 2010 and 2016, the number of inpatient psychiatric beds did not change, but the proportion of those beds operated as for-profit companies and owned by chains increased, with a concentration among freestanding hospitals.^7^ As access to psychiatric treatment in IMD facilities becomes more accessible via public insurance, and calls to eliminate the IMD exclusion proliferate, we may also expect to see an increase in the number of IMDs (ie, freestanding psychiatric hospitals with more than 16 beds) compared with non-IMDs (ie, psychiatric units in general hospitals and small freestanding facilities).^8^ The increasing available funding may result in this increase being concentrated among for-profit facilities.

Given the changes in payment policy, this descriptive study uses national survey data to determine if there have been increases in for-profit ownership among IMD facilities relative to non-IMD facilities, as well as greater increases in Medicaid acceptance among for-profit IMD facilities relative to other facilities.

## Data and methods

We examined changes in ownership and Medicaid acceptance over time among IMD relative to non-IMD facilities using data from the 2014, 2016, 2018, and 2020 versions of the National Mental Health Services Survey (N-MHSS), a census of mental health service delivery data from public and private mental health treatment facilities.^9^ Response rates varied between 88% in 2014 and 90% in 2018.^9-12^ Respondents reported operational information about the facility on April 29 or April 30 of the survey year. While data are available for every year between 2014 and 2020, this study only included survey data from even years, during which facilities were asked, “How many hospital inpatient beds at this facility were specifically designed for providing mental health treatment,” as well as a corresponding question for residential beds.

Cases were deleted from the dataset if they (1) did not provide mental health treatment in a 24-hour hospital inpatient or residential setting, (2) did not accept individuals ages 18–64, and (3) identified as a “residential treatment center for children,” such that the remaining cases reported providing inpatient or residential mental health treatment to adults. Among these, we defined facilities as IMDs if they (1) reported having more than 20 hospital inpatient or residential beds (the survey reports on facilities with 1 to 10 beds, 11 to 20 beds, etc, which prevented us from precisely identifying the cutoff of 17 beds); (2) identified as a “psychiatric hospital,” “residential treatment center for adults,” or “other type of treatment facility”; (3) accepted individuals ages 18–64; and (4) provided mental health treatment in a 24-hour hospital inpatient or residential setting. Because we could not track facilities across surveys using N-MHSS data, each observation represents an individual facility-year. We created indicators for whether each facility accepted Medicaid payment and used the survey-defined ownership variable to identify whether each facility was owned as a private for-profit, private nonprofit, or government (local, state, or federal) facility.

Analyses were descriptive, conducted using Stata 18 (StataCorp). Longitudinal changes in proportional ownership status (private for-profit, private nonprofit, or government) and acceptance of Medicaid were compared between these IMD facilities and the remaining non-IMD facilities. To determine if there was an increase in for-profit ownership among IMD facilities relative to non-IMD facilities, we implemented a multiple logistic regression model with IMD status, year, and the interaction between them as predictors. To determine if there was an increase in Medicaid acceptance among for-profit IMD facilities relative to others, we implemented a logistic regression model with IMD status, ownership status, year, and the interaction between them as predictors of Medicaid acceptance. To account for changes between states during this period, an alternate version of this analysis using state fixed effects is available in the Appendix Table A1 and Figure A1. We also qualitatively described the trends between 2014 and 2020.

## Results

We dropped 34 086 facility-years that did not report an inpatient or residential setting in their facility, an additional 3188 that did not report serving young adults or adults (ages 18–64), and finally, and an additional 113 that specifically identified as a “residential treatment center for children.” Of the remaining 11 139 facility-years, 2773 (23.3%) were IMDs. The IMDs were substantially more likely than non-IMDs to provide mental health treatment in inpatient settings (70.1% vs 54.6%) and slightly less likely to provide treatment in residential settings (43.3% vs 50.2%).

The number of facilities with inpatient and residential beds responding to the N-MHSS decreased by 32.4% between 2014 (*n* = 3320) and 2020 (*n* = 2244). The proportion of these facilities that were IMDs did not change between those years, averaging 23.2% across all years in the study (SD: 1.24 percentage points [pp]).

### Ownership of IMD vs non-IMD facilities

**Figure 1.**
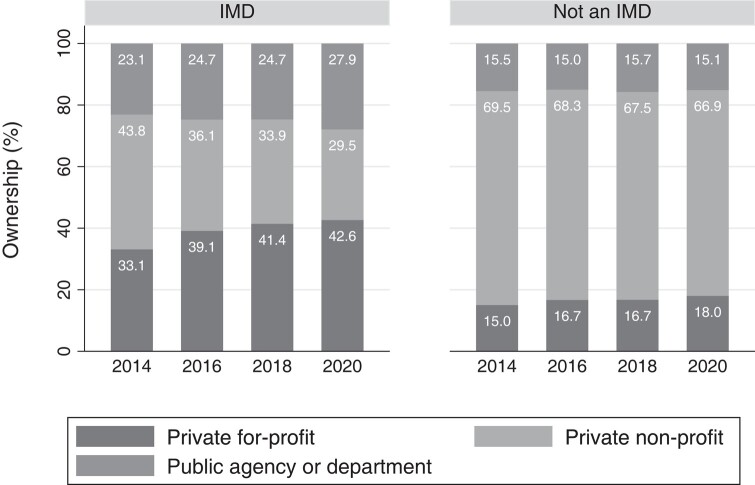
Ownership of inpatient and residential facilities by year and IMD status, 2014–2020. Source: Authors’ analysis of the National Mental Health Services Survey, 2014–2020. *n* = 11 139 facility-years. IMDs are those facilities that reported having more than 20 hospital inpatient or residential beds; identified as a “psychiatric hospital,” “residential treatment center for adults,” or “other type of treatment facility”; accepted individuals ages 18–64; and provided mental health treatment in a 24-hour hospital inpatient or residential setting. Between 2014 and 2020, as laws changed to allow Medicaid payment to IMD settings, ownership of IMDs became more concentrated among for-profits, while non-IMDs did not follow the same pattern and remained primarily nonprofit. Abbreviation: IMD, institution for mental diseases.

**Figure 2.**
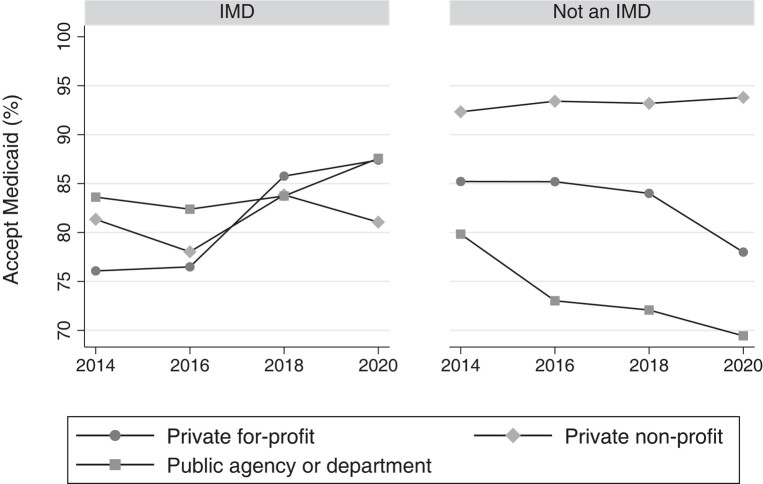
Proportion of inpatient and residential facilities accepting Medicaid by year and IMD status, 2014–2020. Source: Authors’ analysis of the National Mental Health Services Survey, 2014–2020. *n* = 11 139 facility-years. IMDs are those facilities that reported having more than 20 hospital inpatient or residential beds; identified as a “psychiatric hospital,” “residential treatment center for adults,” or “other type of treatment facility”; accepted individuals ages 18–64; and provided mental health treatment in a 24-hour hospital inpatient or residential setting. As laws changed allowing IMDs to allow Medicaid payment to IMD settings between 2014 and 2020, private for-profit IMDs increasingly accepted Medicaid, while the same increase in Medicaid acceptance was not seen among other facility types. Abbreviation: IMD, institution for mental diseases.

The composition of facility ownership changed during this period among both IMD and non-IMD facilities. Among IMD facilities, there was a 9.5-pp increase in ownership by private for-profit organizations between 2014 and 2020 (95% CI: 4.3–5.2 pp; *P* = .000). Among non-IMD facilities, there was a 3.0-pp increase (95% CI: 0.7–6.9 pp; *P* = .001) (Table 1, Figure 1). Relative to non-IMDs, the increase among IMDs was 6.6 pp larger (95% CI: 0.9–12.3 pp; *P* = .024) (Figures 1 and 2). In contrast, nonprofit facilities became a smaller proportion of the composition of IMD facilities (−14.3 pp; 95% CI: −19.4 to −9.2 pp; *P* = .000).

**Table 1.** Changes in IMD and non-IMD facility ownership composition and Medicaid acceptance over time, 2014–2020.

	IMD facilities						Non-IMD facilities					
	2014, %	2016, %	2018, %	2020, %	Change, pp	Change, %	2014, %	2016, %	2018, %	2020, %	Change, pp	Change, %
Ownership composition												
Private for-profit	33.1	39.1	41.4	42.6	9.5	28.7%	15.0	16.7	16.7	18.0	3.0	19.8%
Private nonprofit	43.8	36.1	33.9	29.5	−14.3	−32.7%	69.5	68.3	67.5	66.9	−2.6	−3.8%
Government	23.1	24.7	24.7	27.9	4.8	20.7%	15.4	15.0	15.7	15.1	−0.4	−2.3%
Medicaid acceptance												
All ownership	80.1	78.5	84.6	85.6	5.5	6.8%	89.4	89.1	88.6	87.3	−2.1	0.0%
Private for-profit	76.1	76.5	85.8	87.4	11.3	14.9%	85.2	85.2	84.0	78.0	−7.2	−8.5%
Private nonprofit	81.3	78.0	83.8	81.1	−0.3	−0.3%	92.3	93.4	93.2	93.8	1.5	1.6%
Government	83.6	82.4	83.7	87.6	4.0	4.7%	79.8	73.0	72.1	69.4	−10.4	−13.0%

Source: Authors’ analysis of the National Mental Health Services Survey, 2014–2020. *n* = 11 139 facility-years. IMDs are those facilities that reported having more than 20 hospital inpatient or residential beds; identified as a “psychiatric hospital,” “residential treatment center for adults,” or “other type of treatment facility”; accepted individuals ages 18–64; and provided mental health treatment in a 24-hour hospital inpatient or residential setting. Among IMD facilities, there was a 9.5-percentage point (pp) increase in ownership by private for-profit organizations between 2014 and 2020. Among non-IMD facilities, there was a 3.0-pp increase. Among IMD facilities, the increase in Medicaid acceptance was concentrated among for-profit facilities, which saw an 11.3-pp increase in Medicaid acceptance between 2014 and 2020. Among non-IMD facilities, the decrease is concentrated in for-profit and public facilities.

Abbreviation: IMD, institution for mental diseases.

### Medicaid acceptance among IMD vs non-IMD facilities

Overall acceptance of Medicaid did not change among inpatient and residential mental health facilities, averaging approximately 87.0% among all facility-years. However, Medicaid acceptance did increase slightly among IMD facilities (5.5 pp; 95% CI: 1.4–9.5 pp; *P* = .009) while remaining approximately the same among non-IMD facilities (Table 1, Figure 2).

Among IMD facilities, the increase in Medicaid acceptance was concentrated among for-profit facilities, which saw an 11.3-pp increase in Medicaid acceptance between 2014 and 2020 (95% CI: 4.6–18.0 pp; *P* = .001) compared with no significant change among nonprofit or public facilities. Among non-IMD facilities, the decrease is concentrated in for-profit (−7.2 pp; 95% CI: −13.1 to −1.3 pp; *P* = .017) and public (−10.4 pp; 95% CI: −17.4 to −3.4 pp; *P* = .004) facilities, with no change among nonprofits. As a result, the largest proportional change in Medicaid acceptance occurred among for-profit IMD facilities relative to for-profit non-IMDs (18.5 pp; 95% CI: 9.6–27.5 pp; *P* = .000) (Table 2, Figure 2). While absolute proportions for Medicaid acceptance differed in our alternate analysis using state fixed effects, trends in Medicaid acceptance over time between ownership and facility type remained the same (Appendix Table A1 and Figure A1).

**Table 2.** Changes in facility ownership composition and Medicaid acceptance among IMD facilities relative to non-IMD facilities, 2014–2020.

	Percentage point difference	95% CI	*P*
Ownership composition			
Private for-profit	6.6	(0.9–12.3)	.024*
Private nonprofit	−11.7	(−17.6 to −5.8)	.000*
Government	5.2	(−0.06 to 10.4)	.053
Medicaid acceptance			
Private for-profit	18.5	(9.6–27.5)	.000*
Private nonprofit	−1.8	(−9.3 to 5.8)	.647
Government	14.4	(4.1–24.6)	.006*

Source: Authors’ analysis of the National Mental Health Services Survey, 2014–2020. *n* = 11 139 facility-years. IMDs are those facilities that reported having more than 20 hospital inpatient or residential beds; identified as a “psychiatric hospital,” “residential treatment center for adults,” or “other type of treatment facility”; accepted individuals ages 18–64; and provided mental health treatment in a 24-hour hospital inpatient or residential setting. Between 2014 and 2020, there was a 6.6-percentage point (pp) increase in private for-profit ownership among IMDs relative to non-IMDs, accompanied by an 11.7-pp decrease in nonprofit ownership. In the same years, there was an 18.5 pp increase in Medicaid acceptance among for-profit IMD facilities relative to non-IMD facilities, with no corresponding increase among private nonprofit facilities.

Abbreviation: IMD, institution for mental diseases.

* indicates *P* < .05.

## Discussion

As restrictions loosen on Medicaid reimbursement for IMD facilities, the composition of IMD facilities has become increasingly for-profit relative to non-IMD facilities. Increases in for-profit and government ownership are being counterbalanced by declines in nonprofit ownership, consistent with research on psychiatric facilities from 2010 to 2016.^7^ Medicaid acceptance among for-profit IMDs also increased disproportionately compared with other facilities over time.

Existing research is mixed on the quality of inpatient and residential psychiatric care provided in for-profit vs nonprofit facilities as well as in IMD relative to non-IMD facilities. One study found more episodes of restraint and seclusion, as well as more frequent regulatory complaints, in IMD facilities in Massachusetts compared with non-IMD facilities.^13^ In addition, there is theoretical and empirical evidence that safety and quality may be better in nonprofit vs for-profit hospitals and nursing homes.^14,15^ Our study indicates that non-IMD facilities are predominantly operated as nonprofits, while IMD facilities are much more likely to be operated as for-profit facilities, and these facilities are increasingly accepting Medicaid to pay for care. As for-profit IMD facilities become a larger part of psychiatric patient care both by virtue of their prevalence among inpatient and residential treatment settings and the ability to use Medicaid to pay for them, it will be crucial to understand more about the impacts on care quality.

This study identified a decrease in the total number of inpatient and residential psychiatric facilities, regardless of whether they are IMDs or non-IMDs. We were unable to determine if the decrease in the number of facilities is due to closures or to facility consolidation, which has been occurring rapidly among hospitals in the last decade.^16^ One study noted that, while the number of psychiatric hospitals declined slightly between 2010 and 2016, the number of beds remained relatively stable.^7^ Hospital consolidation has been associated with decreases in quality and may be impacting the quality of care in both IMD- and non–IMD-setting studies in this work.^17,18^

This study had several limitations. First, our definition of an IMD is broad: N-MHSS bed counts are publicly reported in an aggregated fashion (ie, 1 to 10 beds, 11 to 20 beds, 21 to 30 beds, etc), and as such, some IMDs with between 17 and 20 beds may have been classified as non-IMD facilities; however, this would likely bias the results towards an underestimation of true differences between IMD and non-IMD facilities. Second, the N-MHSS does not provide longitudinal facility identifiers, so we were unable to track changes in individual facilities over time. Third, many facilities treat patients in multiple settings. This study assumes that facilities that report accepting Medicaid payments accept these payments for the specific inpatient and residential settings that are relevant to this study. Finally, this is an observational, descriptive study observing changes in ownership and Medicaid acceptance over time, and we are unable to draw causal conclusions.

Calls to repeal the IMD exclusion are increasing.^8,19-22^ Indeed, the Substance Use Disorder Prevention that Promotes Opioid Recovery and Treatment for Patients and Communities (SUPPORT) Act, implemented in 2019, already allows states to pay for SUD treatment in IMD settings for up to 30 days in a year, and evidence suggests that changes in policy via the SUPPORT Act and Section 1115 waivers are impacting SUD treatment in these settings, leading to an increase in the acceptance of Medicaid insurance at facilities that treat SUD and a greater number of for-profit residential facilities.^23,24^ These changes in the SUD space may be having a spillover effect among facilities that treat mental illness. As payment policy increasingly incentivizes for-profits to enter the psychiatric care space, we should be especially mindful of the impact of these decisions on patient safety and quality of care.

## Supplementary Material

qxad089_Supplementary_Data

## References

[1] Musumeci M , Chidambaram P, Orgera K. State Options for Medicaid Coverage of Inpatient Behavioral Health Services. Kaiser Family Foundation; 2019.

[2] Glick ID , Sharfstein SS, Schwartz HI. Inpatient psychiatric care in the 21^st^ century: the need for reform. Psychiatr Serv. 2011;62(2):206–209. 10.1176/ps.62.2.pss6202_0206 21285100

[3] Medicaid Waiver Tracker: Approved and Pending Section 1115 Waivers by State. Kaiser Family Foundation; 2023. Accessed May 17, 2023. https://www.kff.org/medicaid/issue-brief/medicaid-waiver-tracker-approved-and-pending-section-1115-waivers-by-state/

[4] In Lieu of Services and Settings; 2016.

[5] Medicaid and CHIP Payment and Access Commission (MACPAC). Payment for services in institutions for mental diseases (IMDs). Accessed May 17, 2023. https://www.macpac.gov/subtopic/payment-for-services-in-institutions-for-mental-diseases-imds/

[6] Shields MC , Hollander MAG, Marcus SC, Chatterjee P. Characteristics and financing of institutions for mental diseases. Psychiatr Serv. 2021;72(11):1359–1360. 10.1176/appi.ps.202100320 PMC8574225 34734749

[7] Shields MC , Beaulieu ND, Lu S, Busch AB, Cutler DM, Chien AT. Increases in inpatient psychiatry beds operated by systems, for-profits, and chains, 2010–2016. Psychiatr Serv. 2022;73(5):561–564. 10.1176/appi.ps.202100182 34433287 PMC10249908

[8] Glickman A , Sisti DA. Medicaid's institutions for mental diseases (IMD) exclusion rule: a policy debate—argument to repeal the IMD rule. Psychiatr Serv. 2019;70(1):7–10. 10.1176/appi.ps.201800414 30501469

[9] Substance Abuse and Mental Health Services Administration . National Mental Health Services Survey (N-MHSS): 2020. Data on Mental Health Treatment Facilities. Substance Abuse and Mental Health Services Administration; 2021.

[10] Substance Abuse and Mental Health Services Administration . National Mental Health Services Survey (N-MHSS): 2014. Data on Mental Health Treatment Facilities. Substance Abuse and Mental Health Services Administration; 2016. https://www.samhsa.gov/data/sites/default/files/2014_National_Mental_Health_Services_Survey.pdf

[11] Substance Abuse and Mental Health Services Administration . National Mental Health Services Survey (N-MHSS): 2016. Data on Mental Health Treatment Facilities. Substance Abuse and Mental Health Services Administration; 2017. https://www.samhsa.gov/data/sites/default/files/2016_National_Mental_Health_Services_Survey.pdf

[12] Substance Abuse and Mental Health Services Administration . National Mental Health Services Survey (N-MHSS): 2018. Data on Mental Health Treatment Facilities. Substance Abuse and Mental Health Services Administration; 2019. https://www.samhsa.gov/data/sites/default/files/cbhsq-reports/NMHSS-2018.pdf

[13] Shields MC , Hollander MAG. Complaints, restraint, and seclusion in Massachusetts inpatient psychiatric facilities, 2008–2018. J Patient Exp. 2023;10:23743735231179072. 10.1177/23743735231179072 37323757 PMC10265359

[14] Bos A , Boselie P, Trappenburg M. Financial performance, employee well-being, and client well-being in for-profit and not-for-profit nursing homes: a systematic review. Health Care Manage Rev. 2017;42(4):352–368. 10.1097/HMR.0000000000000121 28885990

[15] Chatterjee P , Kelly S, Qi M, Werner RM. Characteristics and quality of US nursing homes reporting cases of coronavirus disease 2019 (COVID-19). JAMA Netw Open. 2020;3(7):e2016930. 10.1001/jamanetworkopen.2020.16930 32725243 PMC8310566

[16] Fulton BD . Health care market concentration trends in the United States: evidence and policy responses. Health Aff (Millwood). 2017;36(9):1530–1538. 10.1377/hlthaff.2017.0556 28874478

[17] Beaulieu ND , Dafny LS, Landon BE, Dalton JB, Kuye I, McWilliams JM. Changes in quality of care after hospital mergers and acquisitions. N Engl J Med. 2020;382(1):51–59. 10.1056/NEJMsa1901383 31893515 PMC7080214

[18] Hayford TB . The impact of hospital mergers on treatment intensity and health outcomes. Health Serv Res. 2012;47(3 Pt 1):1008–1029. 10.1111/j.1475-6773.2011.01351.x 22098308 PMC3423176

[19] Eide S , Gorman CD. Medicaid's IMD Exclusion: The Case for Repeal. The Manhatten Institute; 2021. https://manhattan.institute/article/medicaids-imd-exclusion-the-case-for-repeal

[20] National Alliance on Mental Illness . Medicaid IMD exclusion. Accessed May 22, 2023. https://www.nami.org/Advocacy/Policy-Priorities/Improving-Health/Medicaid-IMD-Exclusion

[21] National Association of State Mental Health Program Directors . Position statement on repeal of the Medicaid IMD exclusion; 2000. Accessed May 22, 2023. https://www.nasmhpd.org/content/position-statement-repeal-medicaid-imd-exclusion

[22] Gray M . States need Congress’ help repealing a law that hinders treatment for mental illness. The Hill. Published January 13, 2022. Accessed May 22, 2023. https://thehill.com/blogs/congress-blog/politics/589703-states-need-congress-help-repealing-a-law-that-hinders-treatment/

[23] Musumeci M , Tolbert J. Federal Legislation to Address the Opioid Crisis: Medicaid Provisions in the SUPPORT Act. Kaiser Family Foundation; 2018. Accessed April 29, 2023. https://www.kff.org/medicaid/issue-brief/federal-legislation-to-address-the-opioid-crisis-medicaid-provisions-in-the-support-act/

[24] Maclean JC , Wen H, Simon KI, Saloner B. Institutions for mental diseases Medicaid waivers: impact on payments for substance use treatment facilities: study examines Medicaid coverage of substance use disorder treatment at residential and outpatient facilities. Health Aff (Millwood). 2021;40(2):326–333. 10.1377/hlthaff.2020.00404 33523735 PMC10161239

