# Supplementary material for: Changes in institution for mental diseases (IMD) ownership status and insurance acceptance over time
Source: Health Aff Sch. 2024 Jan 16;2(1):qxad089. doi: 10.1093/haschl/qxad089 (PMC10790904; doi:10.1093/haschl/qxad089)
Supplement: qxad089_Supplementary_Data [file qxad089_Supplementary_Data.zip › NEW- APPENDIX.docx]

# Appendix

## Table A1: Changes in IMD and non-IMD Medicaid Acceptance Over Time Within States, 2014-2020

|  | ***IMD Facilities*** | | | | | | ***Non-IMD Facilities*** | | | | | |
| --- | --- | --- | --- | --- | --- | --- | --- | --- | --- | --- | --- | --- |
|  | **2014** | **2016** | **2018** | **2020** | **PP Change** | **% Change** | **2014** | **2016** | **2018** | **2020** | **PP Change** | **% Change** |
| **All ownership** | 56.8 | 57.5 | 56.7 | 55.9 | -0.9 | -1.59% | 40.8 | 37.8 | 47.8 | 47.7 | 6.9 | 17.01% |
| **Private for-profit** | 38.0 | 38.3 | 52.9 | 57.6 | 19.6 | 51.6% | 50.0 | 50.0 | 48.3 | 40.0 | -9.97 | -19.9% |
| **Private non-profit or** | 41.4 | 37.2 | 47.6 | 43.4 | 2.0 | 4.7% | 65.1 | 68.6 | 68.0 | 70.3 | 5.19 | 8.0% |
| **Government** | 42.0 | 39.4 | 42.2 | 50.1 | 8.1 | 19.3% | 36.6 | 28.1 | 28.1 | 25.9 | -10.70 | -29.2% |

### TABLE A1

**Caption:** Changes in IMD and non-IMD Medicaid Acceptance Over Time Within States, 2014-2020

**Source/Notes:** Authors’ analysis of the National Mental Health Services Survey, 2014-2020. N=11,139 facility-years. IMDs are those facilities that reported having more than 20 hospital inpatient or residential beds; identified as a “psychiatric hospital,” “residential treatment center for adults,” or “other type of treatment facility”; accepted individuals ages 18-64; and, provided mental health treatment in a 24-hour hospital inpatient or residential setting. Among IMD facilities, the increase in Medicaid acceptance was concentrated among for-profit facilities, which saw a 19.6 pp increase in Medicaid acceptance between 2014 and 2020. Among non-IMD facilities, the decrease is concentrated in for-profit and public facilities.

## Figure A1: Proportion of Inpatient and Residential Facilities Accepting Medicaid by Year and IMD Status, Within State, 2014-2020

##
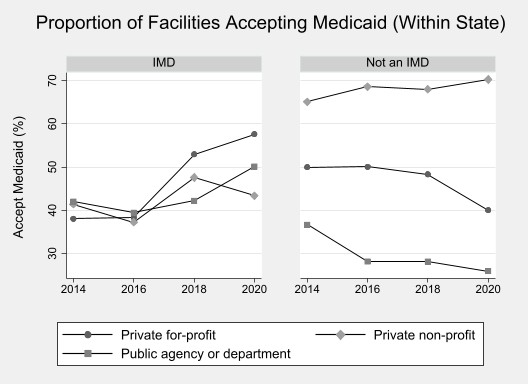


**Source/Notes:** Authors’ analysis of the National Mental Health Services Survey, 2014-2020. N= 11,139 facility-years. IMDs are those facilities that reported having more than 20 hospital inpatient or residential beds; identified as a “psychiatric hospital,” “residential treatment center for adults,” or “other type of treatment facility”; accepted individuals ages 18-64; and, provided mental health treatment in a 24-hour hospital inpatient or residential setting. As laws changed allowing IMDs to allow Medicaid payment to IMD settings between 2014 and 2020, private for-profit IMDs increasingly accepted Medicaid, while the same increase in Medicaid acceptance was not seen among other facility types.
